# Syndrome de Tako-Tsubo associé à une intoxication aiguë aux carbamates en Tunisie : une complication cardiaque rare et multifactorielle

**DOI:** 10.48327/mtsi.v6i2.2026.844

**Published:** 2026-04-20

**Authors:** Sabrine BRADAI, Oussema HADDAR, Yosr BEN TAHER, Mabrouk BAHLOUL

**Affiliations:** Service de réanimation polyvalente, CHU Habib Bourguiba Sfax, 3029, Sfax, Tunisie

**Keywords:** Carbamates, Intoxication, Réanimation, Syndrome de Tako-Tsubo, Cardiomyopathie de stress, Tunisie, Afrique du Nord, Carbamates, Poisoning, Resuscitation, Tako-Tsubo syndrome, Stress cardiomyopathy, Tunisia North Africa

## Abstract

**Introduction:**

Les intoxications aux insecticides de type carbamates anticholinestérasiques peuvent entraîner un tableau grave associant des troubles digestifs, respiratoires et neurologiques. Nous rapportons un cas rare de syndrome de Tako-Tsubo (TTS) survenu en Tunisie de manière concomitante à une intoxication volontaire grave à ce type d’insecticide.

**Observation:**

Une patiente, âgée de 18 ans, a été admise en réanimation pour un coma suite à une ingestion volontaire de méthomyl. Elle présentait initialement des signes cholinergiques. L’évolution a été marquée par l’installation au 2^e^ jour d’un état de choc associé à une crise convulsive. L'échocardiographie a révélé une hypokinésie des segments basaux avec un strain longitudinal global altéré, évoquant un syndrome de TTS inversé. Le bilan biologique a montré des troponines et des taux de NT-proBNP (prohormone N-terminale du peptide natriurétique cérébral) élevés plaidant en faveur du diagnostic. Un contrôle échographique 48 h plus tard a montré une récupération complète de la fonction cardiaque. La patiente a survécu sans séquelles.

**Discussion:**

Le TTS observé après intoxication aux carbamates pourrait résulter d’une triade spasme coronarien, toxicité myo-cardique directe par stress oxydatif et stimulation sympathique excessive, expliquant la dysfonction ventriculaire aiguë.

**Conclusion:**

Ce cas illustre l’implication possible des carbamates dans la genèse d’un TTS via un mécanisme multifactoriel associant déséquilibre autonome et atteinte endothéliale. Il souligne la nécessité d’une vigilance accrue et l’apport déterminant du strain échographique pour affiner le diagnostic et guider la prise en charge.

## Abstract (en)

**Introduction:**

Poisoning by carbamate-type anticholinesterase insecticides can result in severe clinical symptoms involving the gastrointestinal, respiratory, and neurological systems. Here, we present a rare case of Tako-Tsubo syndrome (TTS) that occurred in Tunisia alongside severe intentional poisoning by this type of insecticide.

**Case report:**

An 18-year-old female patient was admitted to the intensive care unit in a coma after intentionally ingesting methomyl. She initially presented with cholinergic symptoms. On the second day, she went into shock and had a seizure. Echocardiography revealed hypokinesis of the basal segments and impaired global longitudinal strain, which is suggestive of reverse TTS. Laboratory tests showed elevated troponin and NT-proBNP levels, which supported the diagnosis. A follow-up echocardiogram 48 hours later showed complete recovery of cardiac function. The patient survived without sequelae.

**Discussion:**

TTS following carbamate poisoning may result from a triad of coronary spasm, direct myocardial toxicity due to oxidative stress, and excessive sympathetic stimulation, which explains the acute ventricular dysfunction.

**Conclusion:**

This case illustrates the possible involvement of carbamates in the pathogenesis of TTS via a multifactorial mechanism combining autonomic imbalance and endothelial damage. The case underscores the need for increased vigilance and the critical role of echocardiographic strain in refining diagnosis and guiding management.

**Figure:**
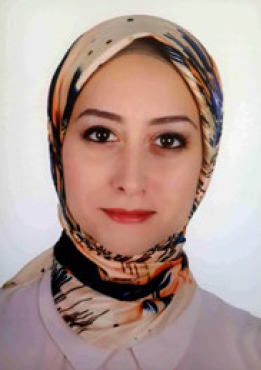

## Introduction

Les carbamates anticholinestérasiques, une classe d’insecticides largement utilisée en agriculture, sont responsables d’intoxications graves, en particulier dans les pays en développement d’Afrique et d’Asie, où leur usage reste souvent insuffisamment réglementé [18].

Bien qu’il n’existe pas d’études épidémiologiques spécifiques évaluant la mortalité liée exclusivement aux carbamates, les intoxications par les pesticides anticholinestérasiques (incluant organophosphorés et carbamates), survenant le plus souvent dans des contextes d’ingestions volontaires ou d’expositions professionnelles accidentelles, sont associées à une mortalité rapportée entre 10 et 20 % dans les formes graves [1].

Bien que leur mécanisme d’action soit réversible, elles peuvent entraîner des complications graves, notamment cardiovasculaires, nécessitant une prise en charge urgente et spécialisée. Parmi ces complications, le syndrome de Tako-Tsubo (TTS), également appelé cardiomyopathie de stress, constitue une entité rare mais grave, caractérisée par une dysfonction transitoire du ventricule gauche, généralement déclenchée par un stress physique ou émotionnel intense [10]. Bien que diverses étiologies toxiques du TTS aient été rapportées, son lien avec une intoxication aux carbamates demeure exceptionnel [5]. À ce jour, un seul cas de TTS secondaire à une intoxication aux carbamates a été rapporté dans la littérature [12]. Nous rapportons ici le deuxième cas documenté de TTS associé à une intoxication grave aux carbamates.

## Observation

Une patiente de 18 ans, sans antécédents, a été admise en réanimation (J1), 2 h après une ingestion volontaire, estimée à environ 30 ml de Lannate contenant 25 % de méthomyl, dans un contexte de conflit familial. À l’examen clinique initial, la patiente avait un score de Glasgow (GCS) à 10/15, était polypnéique à 29 cycles/min, stable sur le plan hémodynamique avec une pression artérielle à 110/70 mmHg et une tachycardie sinusale à 100 battements par minute (bpm). Elle présentait des pupilles en myosis, une hypersialorrhée, des diarrhées et des fasciculations musculaires. Une recherche toxicologique standard a été réalisée à l’admission, les résultats n’étant disponibles qu’après 24 h en raison des délais analytiques. Devant la détresse respiratoire et le risque d’aggravation neurologique, une intubation orotrachéale a été réalisée sous induction par étomidate (0,3 mg/kg) et succinylcholine (1 mg/kg), pour sécuriser les voies aériennes et permettre le lavage gastrique. La sédation a été entretenue par l’association midazolam-fentanyl. La patiente a été mise sous ventilation mécanique invasive en mode contrôlé volumétrique, avec un volume courant de 7 ml/kg, une fréquence respiratoire de 14 cycles/min, une FiO₂ initiale de 50 %, et une PEP de 5 cm H₂O. L’hypersialorrhée a nécessité des aspirations fréquentes, sans argument clinique de bronchospasme.

Un lavage gastrique a été réalisé trois heures après l’ingestion, compte tenu du délai court, de la quantité ingérée et de la gravité potentielle du toxique. Sur la base du tableau clinique évocateur et sans attendre les résultats toxicologiques, un traitement antidotique par pralidoxime méthyl-sulfate (Contrathion) a été initié par voie intraveineuse, avec une dose de charge de 400 mg, suivie de 200 mg toutes les six heures, poursuivie pendant 48 heures.

À l’admission (J1), l’activité cholinestérasique sérique était à 4 252 UI/l. Les marqueurs cardiaques étaient élevés, avec un NT-proBNP (prohormone N-terminale du peptide natriurétique cérébral) à 503 pg/ml et des troponines ultrasensibles à 209 ng/l. L’électrocardiogramme à 12 dérivations initial a objectivé une tachycardie sinusale à 150 bpm, sans trouble du rythme ni anomalie de la conduction.

**Figure 1:**
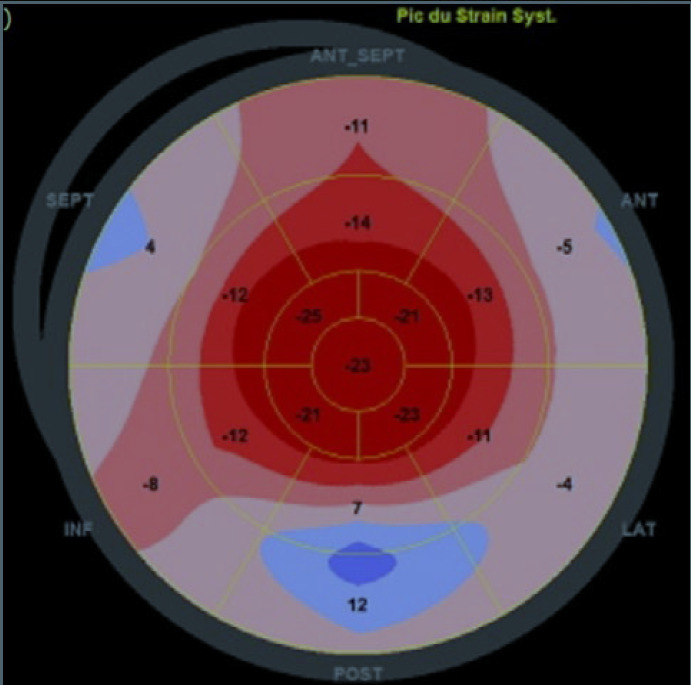
Altération du SLG avec présence de gradient base-apex (aspect en cocarde) à l’échographie cardiaque initiale

Au 2^e^ jour d’hospitalisation (J2), l’évolution a été marquée par l’apparition d’un état de choc, avec une hypotension artérielle à 80/50 mmHg, une tachycardie à 120 bpm et des marbrures, associée à la survenue d’une crise convulsive tonico-clo-nique généralisée, spontanément résolutive. Une échocardiographie transthoracique a été demandée afin d’identifier l’origine du choc. L’examen, réalisé par un cardiologue, a montré un ventricule gauche non dilaté, non hypertrophié, avec une fraction d’éjection ventriculaire gauche à 50 %, associée à une hypokinésie des segments basaux. L’analyse par strain longitudinal global (SLG) a objectivé une altération à 14,5 %, prédominante au niveau des segments basaux, avec un aspect en cocarde, caractérisé par un strain altéré aux bases contrastant avec un strain conservé aux segments apicaux. Ces anomalies échographiques étaient évocatrices d’un TTS inversé (Fig. 1).

Les données biologiques à J2 ont montré une baisse de l’activité cholinestérasique sérique à 2 974 UI/L, une augmentation modérée des troponines à 924 ng/L et une élévation marquée du NT-proBNP à 10 511 pg/ml. Par ailleurs, aucune anomalie électrique n’a été observée sur l’électrocardiogramme. Une perfusion de noradrénaline a été instaurée immédiatement (2 mg/h, sevrée en 72 h).

Le score diagnostique de l’*International Takotsubo Registry* (InterTAK), calculé à J2 à partir des données cliniques et électrocardiographiques disponibles, était de 83 points, confirmant une probabilité très élevée de TTS (Tableau 1I).

**Tableau I:** Le score diagnostique de l’InterTAK calculé chez la patiente

Critère	Présence	Points
Sexe féminin	Oui	25
Stress émotionnel	Oui (conflit familial)	24
Stress physique	Oui (intoxication grave, état de choc)	13
Atteinte neurologique aiguë	Oui (crise convulsive)	9
Absence de sous-décalage ST à l'ECG	Oui	12
Antécédent psychiatrique	Non	0
QTc prolongé	Non	0
**Total**		**83**

**Figure 2:**
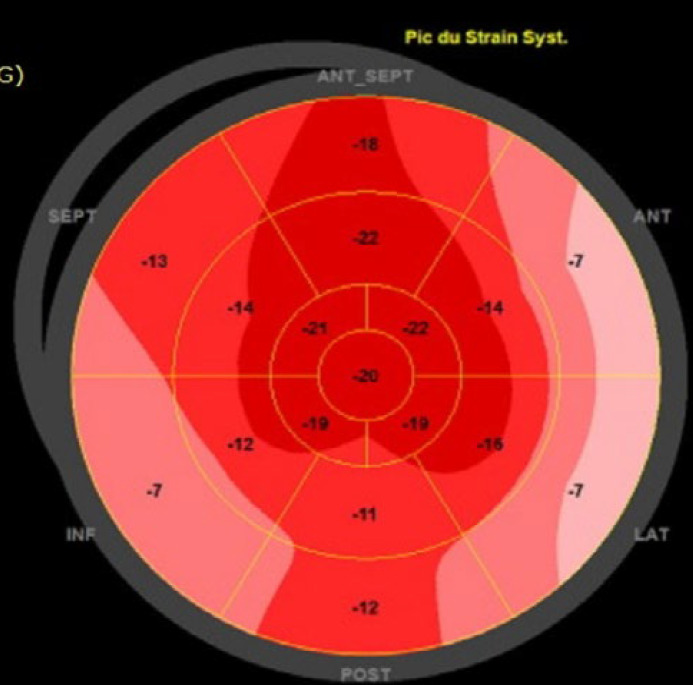
Amélioration du strain myocardique au niveau des segments basaux et médians, 48 h après installation de l’état de choc

Un contrôle échographique avec analyse par strain, réalisé 48 heures plus tard (J4), a montré une récupération de la fonction cardiaque avec une FEVG à 60 %, un SLG redevenu normal à -16,4 %, ainsi qu’une amélioration du strain au niveau des segments basaux et médians (Fig. 2). En se basant sur l’ensemble des données cliniques, biologiques, échographiques et évolutives, le diagnostic de TTS inversé a été retenu chez cette patiente intoxiquée par les carbamates.

La ventilation mécanique invasive a été poursuivie jusqu’au 10^e^ jour d’hospitalisation en raison d’une faiblesse neuromusculaire persistante.

La patiente a récupéré complètement sur le plan neurologique et a été extubée et sevrée de la ventilation mécanique le jour suivant (J11) après rééducation respiratoire. Une prise en charge psychologique a été réalisée avant le retour à domicile, à J13.

## Discussion

À notre connaissance, ce cas est le deuxième rapporté à l’échelle mondiale décrivant une présentation atypique de TTS inversé dans un contexte d’intoxication grave aux carbamates. Le premier cas publié par Lin *et al*. rapportaient également une intoxication grave aux carbamates et pyrethroïdes, caractérisée par un coma profond (GCS à 3), un syndrome cholinergique majeur, une hypokaliémie profonde, et la nécessité d’une intubation et ventilation mécanique [12]. Ce cas clinique souligne l’importance d’approfondir la compréhension des mécanismes physiopathologiques sous-jacents et d’évaluer les implications spécifiques de la prise en charge thérapeutique. Le méthomyl, ou méthyl N-[(méthyl-amino)carbonyl] oxyéthanimidothioate (Lannate, bidon de 20 l), est un carbamate anticholinestérasique utilisé en Tunisie depuis 1976. Bien qu’il soit couramment utilisé en milieu professionnel à visée insecticide, il est également largement utilisé à des fins domestiques. Les carbamates, progressivement substitués aux organochlorés, sont privilégiés pour leur faible rémanence environnementale et leur absence de bioaccumulation dans la chaîne alimentaire [2]. Cependant, leur accessibilité en vente libre et leur faible coût en font un produit fréquemment utilisé lors de tentatives d’autolyse, notamment chez les jeunes adultes. Une étude rétrospective menée au Centre d’assistance médicale urgente (CAMU) de Tunis, entre 2009 et 2012, a révélé que la majorité des intoxications aux carbamates étaient volontaires, survenant principalement chez des femmes jeunes, avec une mortalité hospitalière de 13 % [2].

Le tableau clinique classiquement décrit lors des intoxications aux carbamates résulte de l’inhibition réversible de l’acétylcholinestérase, responsable d’une accumulation d’acétylcholine au niveau des synapses cholinergiques. Le tableau clinique associe des signes muscariniques (hypersalivation, myosis, bradycardie, bronchorrhée, diarrhée, vomissements), des signes nicotiniques (fasciculations, faiblesse musculaire, paralysie respiratoire) et des manifestations centrales (agitation, confusion, convulsions, coma) [2].

Le diagnostic de TTS a été retenu sur la base d’arguments cliniques, biologiques, échocar-diographiques et évolutifs concordants. Le TTS, décrit pour la première fois en 1990 par Hikaru Sato, correspond à une cardiomyopathie transitoire caractérisée par une dysfonction réversible du ventricule gauche, le plus souvent déclenchée par un stress physique ou émotionnel intense [6]. Selon les recommandations de l’*European society of cardiology*, le diagnostic repose sur la mise en évidence d’une altération transitoire de la fonction systolique du ventricule gauche en l’absence de lésion coronarienne significative. Les anomalies électrocardiographiques sont fréquentes mais non constantes, et le profil biologique associe une élévation modérée des troponines à une augmentation marquée du NT-proBNP, comme observé chez notre patiente [6].

L’échocardiographie transthoracique est l’examen de première intention pour le diagnostic du TTS, car elle permet d’évaluer la fonction systolique du ventricule gauche et de détecter les anomalies segmentaires du mouvement pariétal [3]. Cependant, certaines formes atypiques, telles que les variantes inversées ou médio-ventriculaires, peuvent échapper à une analyse conventionnelle. Dans ce contexte, le strain myocardique, une technique avancée d’échocardiographie, constitue un outil diagnostique utile. Il mesure la déformation longitudinale du myocarde au cours du cycle cardiaque, fournissant une évaluation sensible et régionale de la fonction myocardique. En cas de TTS, le strain met en évidence un gradient base-apex et des altérations de la déformation touchant plusieurs segments, répartis de manière non systématisée, c’est-à-dire dépassant le territoire d’une seule artère coronaire, à la différence des syndromes coronariens aigus [4,9]. Le strain permet ainsi non seulement d’affiner le diagnostic et de typifier la variante anatomique, mais aussi de suivre l’évolution de la récupération myocardique. La physiopathologie du TTS reste incomplètement élucidée, mais l’hyperactivation du système sympathique et la libération excessive de catécho-lamines jouent un rôle central, associées à des facteurs prédisposants tels qu’une susceptibilité hormonale ou des antécédents psychiatriques [6]. À la lumière de ces éléments, nous proposons désormais des hypothèses pour expliquer la physiopathologie du TTS en cas d›intoxication aux carbamates.

D’abord, le spasme coronarien épicardique et microvasculaire transitoire, récemment identifié comme un mécanisme pathogénique probable du TTS [5,14], pourrait être exacerbé par l’effet des carbamates en raison de leur interaction avec le système cholinergique, particulièrement chez un patient présentant un dysfonctionnement endothélial transitoire [11,15,16].

En effet, les carbamates, en inhibant l’acétylcholinestérase, augmentent les concentrations d’acétylcholine dans la synapse, intensifiant l’activation des récepteurs muscariniques M3 présents sur les cellules musculaires lisses vasculaires. Dans des conditions normales, l’activation des récepteurs M3 par l’acétylcholine favorise la libération de monoxyde d’azote (NO) par l’endothélium fonctionnel, induisant une vasodilatation *via* la voie de la GMPc (guanosine monophosphate cyclique). Toutefois, en cas de dysfonctionnement endothélial suite à un stress aigu ou chronique, l’activation des récepteurs M3 engendre une surcharge calcique intracellulaire dans les cellules musculaires lisses vasculaires, ce qui active la calmoduline kinase. Cette activation induit la phosphorylation de la chaîne légère de la myosine, entraînant une constriction coronarienne intense [19]. Ainsi, l’exposition aux carbamates peut amplifier ce mécanisme en augmentant la stimulation cholinergique, contribuant au spasme coronarien épicardique et microvasculaire.

Le 2^e^ mécanisme physiopathologique envisagé concerne la toxicité directe des carbamates sur les cardiomyocytes, *via* la production de radicaux libres et le stress oxydatif. Les carbamates ont été identifiés comme des inducteurs de dysfonctionnement mitochondrial, d’inflammation et de stress oxydatif [7]. Ce dernier contribue à l’augmentation de la perméabilité vasculaire, favorise l’adhésion des leucocytes et perturbe la transduction du signal endothélial [17]. De plus, YS Jung *et al*. ont rapporté que les carbamates ont induit la mort cellulaire par apoptose dans les cellules endothéliales microvasculaires cérébrales [8], ce qui pourrait expliquer les manifestations neurologiques, telles que la crise convulsive observée chez notre patiente. Ce mécanisme de toxicité endothéliale pourrait également être impliqué au niveau des artères coronaires, favorisant une dysfonction microvasculaire susceptible de contribuer à la genèse du TTS.

**Figure 3:**
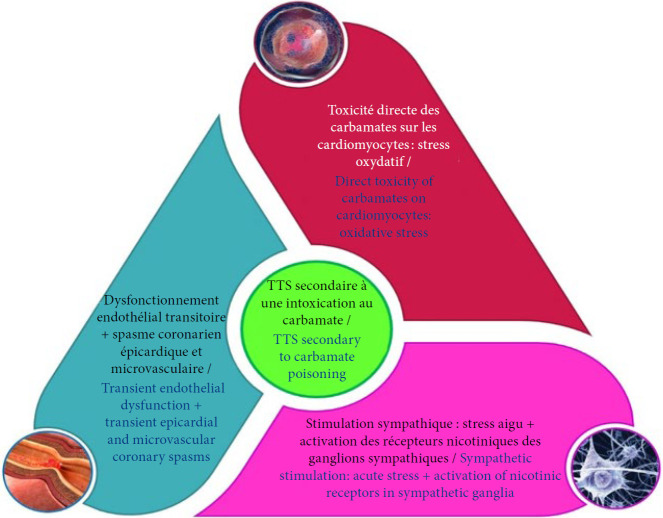
Triade physiopathologique du TTS secondaire à une intoxication aux carbamates

Le 3^e^ mécanisme physiopathologique envisagé repose sur une stimulation sympathique multifactorielle. Cette stimulation est d›abord déclenchée par un stress aigu et des troubles psychiatriques préexistants avant l›intoxication, ce qui favorise l›hyper-contraction coronarienne [13]. Par ailleurs, le carbamate induit une toxicité cholinergique en inhibant l’acétylcholinestérase, entraînant une accumulation d’acétylcholine au niveau des synapses neuronales et de la jonction neuromusculaire. L’activation des récepteurs nicotiniques dans les ganglions sympathiques peut entraîner des effets adrénergiques, tels que la tachycardie, l’hypertension et la mydriase [13], contribuant ainsi à la stimulation sympathique. Nous proposons une triade qui résume la physiopathologie du TTS secondaire à une intoxication au carbamate (Fig. 3).

La prise en charge du TTS, en particulier dans le cadre d’une intoxication aux carbamates, doit être rapide et ciblée. Chez notre patiente, la prise en charge a combiné le traitement du choc cardiogénique et celui de l’intoxication cholinergique. Le méthylsulfate de pralidoxime (Contrathion) a été administré précocement, compte tenu de la sévérité du tableau et de la possibilité d’une intoxication associée aux organophosphorés. Malgré une efficacité controversée dans les intoxications aux carbamates, son utilisation visait à restaurer l’activité cholinestérasique et à limiter la surcharge cholinergique. L’atropine n’a pas été utilisée en raison de la dysfonction myocardique, son effet chronotrope positif pouvant majorer la consommation myocardique en oxygène. Un traitement par noradrénaline intraveineuse a été instauré pour corriger l’hypotension persistante et maintenir une pression artérielle moyenne ≥ 65 mmHg. Cette approche thérapeutique combinée a permis non seulement de gérer les manifestations cliniques du TTS, mais aussi de traiter la cause sous-jacente de l’intoxication, facilitant ainsi une récupération plus rapide et complète de la fonction cardiaque.

## Conclusion

Ce cas illustre la nécessité de considérer le TTS parmi les complications possibles des intoxications aiguës aux carbamates, en particulier devant un état de choc inexpliqué. Il souligne la valeur diagnostique du strain myocardique dans la détection précoce et le suivi de la dysfonction ventriculaire transitoire. Au-delà de l’intérêt clinique, cette observation met en évidence la complexité physiopathologique du lien entre toxicité cholinergique et atteinte myocardique. Enfin, dans le contexte tunisien, la large accessibilité des carbamates impose de renforcer les mesures de régulation et de sensibilisation afin de prévenir les intoxications volontaires et leurs complications cardiovasculaires parfois graves.

## Contribution des auteurs et autrices

Sabrine Bradai : conception, rédaction, révision et validation du manuscrit.

Oussema Haddar : conception, rédaction et révision du manuscrit.

Yosr ben Taher : rédaction et révision du manuscrit.

Mabrouk Bahloul : rédaction, révision et validation du manuscrit.

## Conflits d’intérêts et principes éthiques

Aucun conflit d’intérêt lié à ce travail n’a été déclaré. Nous n’avons pas de financement lié à ce travail.

## Remerciements

Nous tenons à exprimer notre profonde gratitude pour leurs contributions à la rédaction et la révision de cet article au Professeur Chokri Ben Hamida, chef du service de réanimation médicale du CHU Habib Bourguiba, et au Professeur Laila Abid, cheffe du service de cardiologie du CHU Hedi Chaker.
