# Supplementary material for: Tako-Tsubo syndrome associated with acute carbamate poisoning in Tunisia: a rare, multifactorial cardiac complication
Source: Med Trop Sante Int. 2026 Apr 20;6(2):mtsi.v6i2.2026.844. [Article in French] doi: 10.48327/mtsi.v6i2.2026.844 (PMC13423296; doi:10.48327/mtsi.v6i2.2026.844)
Supplement: Related texts

# Research article: Tako-Tsubo syndrome associated with acute carbamate poisoning in Tunisia: a rare, multifactorial cardiac complication

## Introduction

Anticholinesterase carbamates are a class of insecticides that are widely used in agriculture and are responsible for serious poisonings, particularly in developing countries in Africa and Asia, where their use is often insufficiently regulated [18].

While there are no epidemiological studies exclusively assessing mortality linked to carbamates, poisonings from anticholinesterase pesticides (including organophosphates and carbamates) are associated with a reported mortality rate of 10-20% in severe cases of intentional ingestion or accidental occupational exposure [1].

Their mechanism of action is reversible, but they can lead to severe complications, particularly cardiovascular ones, requiring urgent, specialized care. One such complication is Tako-Tsubo syndrome (TTS), also known as stress cardiomyopathy. This rare but severe condition is characterized by transient left ventricular dysfunction and is typically triggered by intense physical or emotional stress [10]. While various toxic etiologies of TTS have been reported, its association with carbamate poisoning is rare [5]. To date, only one case of TTS secondary to carbamate poisoning has been reported in the literature [12].

Here, we report the second documented case of TTS associated with severe carbamate poisoning.

## Case report

An 18-year-old female patient with no prior medical history was admitted to the intensive care unit on Day 1 (D1), two hours after voluntarily ingesting an estimated 30 mL of Lannate, which contains 25% methomyl. The ingestion occurred following a family dispute. Initial clinical examination revealed a Glasgow Coma Scale (GCS) score of 10/15, tachypnea at 29 breaths per minute, hemodynamic stability with a blood pressure of 110/70 mmHg, and sinus tachycardia at 100 beats per minute (bpm). The patient presented with miosis, hypersalivation, diarrhea, and muscle fasciculations. Standard toxicology testing was performed upon admission, but the results were not available for 24 hours due to laboratory turnaround times.

Due to respiratory distress and the risk of neurological deterioration, orotracheal intubation was performed with etomidate (0.3 mg/kg) and succinylcholine (1 mg/kg) induction to secure the airway and enable gastric lavage. Sedation was maintained with midazolam and fentanyl. The patient was placed on invasive mechanical ventilation in volume-controlled mode with a tidal volume of 7 mL/kg, a respiratory rate of 14 breaths per minute, an initial fraction of inspired oxygen (FiO₂) of 50%, and a positive end-expiratory pressure (PEEP) of 5 cm H₂O. Hypersalivation required frequent suctioning with no evidence of bronchospasm.

Gastric lavage was performed three hours after ingestion due to the short time elapsed, the amount ingested, and the potential severity of the toxin. Based on the suggestive clinical presentation, antidote therapy with pralidoxime methylsulfate (Contrathion) was initiated intravenously without waiting for toxicological results. The initial dose was 400 mg, followed by 200 mg every six hours for 48 hours.

Upon admission on D1, serum cholinesterase activity was measured at 4,252 IU/L. Elevated cardiac markers were present, including an NT-proBNP level of 503 pg/mL and high-sensitivity troponin level of 209 ng/L. The initial 12-lead electrocardiogram revealed sinus tachycardia at 150 beats per minute (bpm), with no arrhythmia or conduction abnormalities.

On the second day (D2) of hospitalization, the patient’s condition deteriorated with the onset of shock, characterized by arterial hypotension at 80/50 mmHg, tachycardia at 120 beats per minute (bpm), and mottling. The patient also experienced a generalized tonic-clonic seizure that resolved spontaneously. A transthoracic echocardiogram was ordered to identify the cause of the shock. The cardiologist who performed the examination revealed a non-dilated, non-hypertrophied left ventricle with a left ventricular ejection fraction of 50%, associated with hypokinesis of the basal segments. Global longitudinal strain (GLS) analysis revealed a reduction of −14.5%, predominantly in the basal segments, with a rosette pattern, which is characterized by reduced strain at the bases that contrasts with preserved strain in the apical segments. These echocardiographic abnormalities were suggestive of inverted TTS (Fig. 1).

**Figure 1:** Lesion of the GLS with a base-to-apex gradient (rosette pattern) on the initial echocardiogram

Laboratory data on day two showed decreased serum cholinesterase activity at 2,974 IU/L, a moderate increase in troponins at 924 ng/L, and a significant increase in NT-proBNP at 10,511 pg/mL. Additionally, no electrical abnormalities were observed on the electrocardiogram. A noradrenaline infusion was initiated immediately (2 mg/h, tapered off over 72 hours).

The International Takotsubo Registry (InterTAK) diagnostic score, calculated on D2 based on available clinical and electrocardiographic data, was 83 points, confirming a very high probability of TTS (Table 1I).

**Table I:** InterTAK diagnostic score calculated for the patient

Criterion	Present	Points
Female	Yes	25
Emotional stress	Yes (family conflict)	24
Physical stress	Yes (severe poisoning, shock)	13
Acute neurological impairment	Yes (seizure)	9
Absence of ST-segment depression on the ECG	Yes	12
History of psychiatric disorders	No	0
Prolonged QTc	No	0
**Total**		**83**

Forty-eight hours later (D4), an echocardiogram with strain analysis showed recovery of cardiac function, with an LVEF of 60%, a normalized LVST of -16.4%, and improved strain in the basal and mid-segments (Fig. 2).

**Figure 2:** Improved myocardial strain in the basal and mid-segments, 48 hours after the onset of shock

A diagnosis of reverse TTS was established in this patient with carbamate poisoning based on combined clinical, laboratory, echocardiographic, and clinical course data.

Invasive mechanical ventilation continued until the tenth day of hospitalization due to persistent neuromuscular weakness.

The patient made a complete neurological recovery, was extubated, and was weaned from mechanical ventilation the following day (D11) after undergoing respiratory rehabilitation. Psychological counseling was provided prior to discharge on D13.

## Discussion

To our knowledge, this is the second reported case worldwide of an atypical presentation of reverse TTS in the context of severe carbamate poisoning. The first case, published by Lin *et al.*, involved severe carbamate and pyrethroid poisoning. It was characterized by deep coma (GCS 3), major cholinergic syndrome, severe hypokalemia, and the need for intubation and mechanical ventilation [12]. This case emphasizes the need to improve our understanding of the underlying pathophysiological mechanisms and to evaluate the implications for therapeutic management.

Methomyl (Lannate, 20-liter container), also known as methyl N-[(methylamino)carbonyl] oxyethanimidothioate, is an anticholinesterase carbamate that has been used in Tunisia since 1976. It is commonly used in professional settings as an insecticide but is also widely used for domestic purposes. Carbamates have gradually replaced organochlorines due to their low environmental persistence and lack of bioaccumulation in the food chain [2]. However, their over-the-counter availability and low cost make them frequently used in suicide attempts, particularly among young adults. A retrospective study conducted at the Emergency Medical Assistance Center (CAMU) in Tunis from 2009 to 2012 revealed that most carbamate poisonings were intentional and primarily occurred in young women. The hospital mortality rate was 13% [2].

The clinical picture typically associated with carbamate poisoning results from reversible inhibition of acetylcholinesterase, which leads to an accumulation of acetylcholine at cholinergic synapses. This combination of signs includes muscarinic symptoms such as hypersalivation, miosis, bradycardia, bronchorrhea, diarrhea, and vomiting; nicotinic symptoms such as fasciculations, muscle weakness, and respiratory paralysis; and central nervous system symptoms such as agitation, confusion, convulsions, and coma [2]. The diagnosis of TTS was made based on concordant clinical, laboratory, echocardiographic, and course of illness findings. First described in 1990 by Hikaru Sato, TTS is a transient cardiomyopathy characterized by reversible left ventricular dysfunction, most often triggered by intense physical or emotional stress [6]. According to the European Society of Cardiology’s recommendations, the diagnosis is based on demonstrating transient impairment of left ventricular systolic function in the absence of significant coronary artery disease. Electrocardiographic abnormalities are common but not constant. The laboratory profile shows moderate troponin elevation combined with a significant NT-proBNP increase, as observed in our patient [6].

Transthoracic echocardiography is the primary test used to diagnose TTS because it enables the evaluation of left ventricular systolic function and the identification of abnormalities in wall motion [3]. However, certain atypical forms, such as inverted or mid-ventricular variants, may escape conventional analysis. In these cases, myocardial strain, an advanced echocardiographic technique, is a useful diagnostic tool. It measures longitudinal myocardial deformation during the cardiac cycle, providing a sensitive regional assessment of myocardial function. In cases of TTS, strain reveals a base-to-apex gradient and alterations in deformation affecting multiple, non-systematically distributed segments—unlike acute coronary syndromes [4,9]. Thus, strain allows for refining the diagnosis, characterizing the anatomical variant, and monitoring the progression of myocardial recovery.

The pathophysiology of TTS is not fully understood, but hyperactivation of the sympathetic nervous system and excessive release of catecholamines play a central role. This is associated with predisposing factors, such as hormonal susceptibility or a history of psychiatric disorders [6]. In light of these findings, we propose the following hypotheses to explain the pathophysiology of TTS in cases of carbamate poisoning.

First, transient epicardial and microvascular coronary spasms, which have been identified as a potential mechanism of TTS [5,14], could be exacerbated by carbamate interaction with the cholinergic system, particularly in patients with transient endothelial dysfunction [11,15,16].

By inhibiting acetylcholinesterase, carbamates increase acetylcholine concentrations in the synapse, thereby intensifying the activation of M3 muscarinic receptors on vascular smooth muscle cells. Under normal conditions, activation of these receptors by acetylcholine promotes nitric oxide (NO) release by the functional endothelium, inducing vasodilation via the cGMP pathway. However, in the event of endothelial dysfunction following acute or chronic stress, activation of the M3 receptors leads to an intracellular calcium overload in the vascular smooth muscle cells, activating the calmodulin-dependent protein kinase. This induces the phosphorylation of the myosin light chain, resulting in intense coronary constriction [19]. Therefore, exposure to carbamates can amplify this mechanism by increasing cholinergic stimulation and contributing to epicardial and microvascular coronary spasms. The second pathophysiological mechanism involves the direct toxicity of carbamates on cardiomyocytes via free radical production and oxidative stress. Carbamates have been identified as inducers of mitochondrial dysfunction, inflammation, and oxidative stress [7]. The latter contributes to increased vascular permeability, promotes leukocyte adhesion, and disrupts endothelial signal transduction [17]. Furthermore, YS Jung *et al*. reported that carbamates induce apoptosis in cerebral microvascular endothelial cells [8], which could explain the seizure observed in our patient and other neurological manifestations. This mechanism of endothelial toxicity may also affect the coronary arteries, promoting microvascular dysfunction that could contribute to the development of TTS.

The third proposed pathophysiological mechanism is based on multifactorial sympathetic stimulation. This stimulation is initially triggered by acute stress and pre-existing psychiatric disorders before poisoning occurs, promoting coronary hypercontraction [13]. Furthermore, carbamates induce cholinergic toxicity by inhibiting acetylcholinesterase, which leads to an accumulation of acetylcholine at neuronal synapses and the neuromuscular junction. Activation of nicotinic receptors in the sympathetic ganglia can lead to adrenergic effects, such as tachycardia, hypertension, and mydriasis, thereby contributing to sympathetic stimulation [13].

We propose a triad that summarizes the pathophysiology of TTS secondary to carbamate poisoning (Fig. 3).

**Figure 3:** Pathophysiological triad of TTS secondary to carbamate poisoning

The management of TTS, particularly in cases of carbamate poisoning, requires a rapid and targeted approach. For our patient, treatment for cardiogenic shock was combined with treatment for cholinergic poisoning. Given the severity of the clinical presentation and the possibility of concomitant organophosphate poisoning, pralidoxime methyl sulfate (Contrathion) was administered early. Despite the controversy surrounding its efficacy in carbamate poisonings, it was used to restore cholinesterase activity and limit cholinergic overload. Atropine was not used due to myocardial dysfunction because its positive chronotropic effect could increase myocardial oxygen consumption. We initiated treatment with intravenous norepinephrine to correct persistent hypotension and maintain a mean arterial pressure of at least 65 mmHg. This combined therapeutic approach managed the clinical manifestations of TTS and treated the underlying cause of the poisoning. This facilitated a faster and more complete recovery of cardiac function.

## Conclusion

This case highlights the importance of considering TTS as a possible complication of acute carbamate poisoning, especially when unexplained shock is present. It also emphasizes the diagnostic value of myocardial strain for the early detection and monitoring of transient ventricular dysfunction. Beyond its clinical relevance, this case emphasizes the pathophysiological complexity of the relationship between cholinergic toxicity and myocardial damage. In the Tunisian context, where carbamates are widely available, strengthening regulatory measures and public awareness efforts is crucial to prevent intentional poisoning and its severe cardiovascular complications.

## Authors’ contributions

Sabrine Bradai: conception, drafting, revision, and validation of the manuscript.

Oussema Haddar: conception, drafting, and revision of the manuscript.

Yosr Ben Taher: Drafting and revising the manuscript.

Mabrouk Bahloul: Drafting, revising, and validating the manuscript.

## Conflicts of Interest and Ethical Principles

No conflicts of interest related to this work were reported. We received no funding related to this work.

## Acknowledgments

We would like to express our deepest gratitude to Professor Chokri Ben Hamida, Head of the Medical Intensive Care Unit at Habib Bourguiba University Hospital, and to Professor Laila Abid, Head of the Cardiology Department at Hedi Chaker University Hospital, for their contributions to drafting and revising this article.
